# *In silico* Prediction of Virus-Host Interactions for Marine Bacteroidetes With the Use of Metagenome-Assembled Genomes

**DOI:** 10.3389/fmicb.2020.00738

**Published:** 2020-04-28

**Authors:** Kento Tominaga, Daichi Morimoto, Yosuke Nishimura, Hiroyuki Ogata, Takashi Yoshida

**Affiliations:** ^1^Laboratory of Marine Microbiology, Division of Applied Biosciences, Graduate School of Agriculture, Kyoto University, Kyoto, Japan; ^2^Laboratory of Marine Environmental Microbiology, Division of Applied Biosciences, Graduate School of Agriculture, Kyoto University, Kyoto, Japan; ^3^Atmosphere and Ocean Research Institute, The University of Tokyo, Kashiwa, Japan; ^4^Chemical Life Science, Bioinformatics Center, Institute for Chemical Research, Kyoto University, Uji, Japan

**Keywords:** Bacteroidetes, Bacteroidetes virus, environmental viral genomes, computational viral host prediction, metagenome assembled genomes

## Abstract

Bacteroidetes is one of the most abundant heterotrophic bacterial taxa in the ocean and play crucial roles in recycling phytoplankton-derived organic matter. Viruses of Bacteroidetes are also expected to have an important role in the regulation of host communities. However, knowledge on marine Bacteroidetes viruses is biased toward cultured viruses from a few species, mainly fish pathogens or Bacteroidetes not abundant in marine environments. In this study, we investigated the recently reported 1,811 marine viral genomes to identify putative Bacteroidetes viruses using various *in silico* host prediction techniques. Notably, we used microbial metagenome-assembled genomes (MAGs) to augment the marine Bacteroidetes reference genomic data. The examined viral genomes and MAGs were derived from simultaneously collected samples. Using nucleotide sequence similarity-based host prediction methods, we detected 31 putative Bacteroidetes viral genomes. The MAG-based method substantially enhanced the predictions (26 viruses) when compared with the method that is solely based on the reference genomes from NCBI RefSeq (7 viruses). Previously unrecognized genus-level groups of Bacteroidetes viruses were detected only by the MAG-based method. We also developed a host prediction method based on the proportion of Bacteroidetes homologs in viral genomes, which detected 321 putative Bacteroidetes virus genomes including 81 that were newly recognized as Bacteroidetes virus genomes. The majority of putative Bacteroidetes viruses were detected based on the proportion of Bacteroidetes homologs in both RefSeq and MAGs; however, some were detected in only one of the two datasets. Putative Bacteroidetes virus lineages included not only relatives of known viruses but also those phylogenetically distant from the cultured viruses, such as marine Far-T4 like viruses known to be widespread in aquatic environments. Our MAG and protein homology-based host prediction approaches enhanced the existing knowledge on the diversity of Bacteroidetes viruses and their potential interaction with their hosts in marine environments.

## Introduction

Marine heterotrophic prokaryotes are responsible for processing almost half of the organic matter that is fixed by marine phytoplankton, thus playing an important role in the global carbon cycle (Azam and Malfatti, 2007). Members of the phylum Bacteroidetes are the most abundant heterotrophic prokaryotes in the ocean along with those belonging to Proteobacteria (Glöckner et al., 1999; Kirchman, 2002). Bacteroidetes inhabit various marine environments ranging from coastal water to open ocean habitats (Alonso et al., 2007; Pommier et al., 2007). They are especially abundant during and after the phytoplankton blooms and believed to have an important role in the decomposition and remineralization of the phytoplankton biomass (Teeling et al., 2012). A previous study suggests that there are 1,200 species of marine planktonic Bacteroidetes and only about half of their global diversity has been described by cultivation (Alonso et al., 2007). Despite being the abundant species during phytoplankton blooms, isolated marine Bacteroidetes strains are rarely observed in environment; therefore, most abundant lineages of marine Bacteroidetes remain poorly characterized (Unfried et al., 2018).

Marine viruses are being increasingly recognized as important factors affecting the structure and function of the prokaryotic community through diverse virus-host interactions, which drive the global biochemical cycle in the ocean (Suttle, 2007; Yoshida et al., 2019). Considering the importance of Bacteroidetes in the marine biochemical cycle, their viruses also likely have significant impact on the process. To date, 58 genomes have been reported for Bacteroidetes viruses isolated from aquatic environments (Puig and Girones, 1999; Borriss et al., 2007; Cheng et al., 2012; Kang et al., 2012a, b, 2015, 2016; Holmfeldt et al., 2013; Castillo et al., 2014; Luhtanen et al., 2014; Laanto et al., 2015; Castillo and Middelboe, 2016; Mihara et al., 2016). However, their hosts cover only seven species of Bacteroidetes. Moreover, the host species of these viruses were biased toward coastal rare taxa (e.g., *Cellulophaga baltica*) or fish pathogen *Flavobacterium*. Therefore, our understanding on marine Bacteroidetes viruses brought by cultivation-based approaches are limited to less abundant taxa in the ocean.

Owing to the recent development of sequencing technology, viral metagenomes (viromes) have become a powerful tool to characterize the diversity of viruses as an alternative of the classical cultivation strategy (Brum and Sullivan, 2015). For example, Nishimura et al. (2017a) recently constructed 1,600 complete environmental viral genomes (EVGs) from marine viromes. Among them, the authors identified 239 viral genomes which were classified into two groups, referred to as groups 1 and 2, likely infecting *Flavobacteriaceae*, a major group of marine Bacteroidetes (Nishimura et al., 2017a). Although these groups include highly diverse viruses (representing 29 and 25 genus-level OTUs (gOTUs) based on genomic similarity), they showed a significant genomic similarity with the cultured siphoviruses infecting *Non-labens* (group 1) or the podovirus phi38:1 infecting *C. baltica* (group 2; one of the most globally abundant type of virus in the oceans), respectively (Roux et al., 2016; Nishimura et al., 2017a). Thus, our knowledge of the genome repertoire of marine Bacteroidetes viruses are still limited to the relatives of cultured Bacteroidetes viruses even after the application of viral metagenomics approaches.

Since viromes revealed enormous diversity of viruses with no isolated relatives, linking these viruses with their putative hosts by culture independent methods has become important to gain insights into the ecology of viruses. Recently, several *in silico* host prediction approaches using viral and microbial genomes have been developed (Edwards et al., 2016; Ahlgren et al., 2017). These methods detect virus-host signals in viral and microbial genomes, which are shaped by virus-host co-evolutionary processes such as acquisition of CRIPSR spacer sequences (Edwards et al., 2016). However, genomic information of uncultured microorganisms is still limited (Rappé and Giovannoni, 2003; Locey and Lennon, 2016) and represents a major hurdle to expand our knowledge of virus-host interaction even though such *in silico* approaches.

Recently, metagenome assembled genomes (MAGs), which can aid us in overcoming this limitation, are receiving increasing attention. Development of metagenomic assembly, binning, and curation techniques have enabled us to construct nearly complete genomes of uncultured microorganisms from various environments (Anantharaman et al., 2016; Bowers et al., 2017; Parks et al., 2017; Tully et al., 2017, 2018; Delmont et al., 2018; Stewart et al., 2018; Almeida et al., 2019; Nayfach et al., 2019; Pasolli et al., 2019). Recent studies have reported over 3000 microbial MAGs including over 500 putative Bacteroidetes MAGs (Tully et al., 2017, 2018; Delmont et al., 2018) from metagenomic samples obtained from the *Tara* Oceans expedition (Sunagawa et al., 2015).

In this study, we performed a computational host prediction analysis for a thousand of EVGs, using the Bacteroidetes MAGs as potential host genomes, to overcome the bottleneck of viral host prediction and expand our knowledge of the diversity of Bacteroidetes viruses. The MAG based prediction approach is expected to detect lineage-specific interactions between EVGs and their hosts, which will be compared with the previous family level host prediction of *Flavobacteriaceae* EVG group 1 and 2. Considering the locality of marine virus-host interaction (Brum et al., 2015; Yoshida et al., 2018), these microbial MAGs likely represent ideal host candidates for the EVGs, because most of the MAGs and EVGs were obtained from simultaneously sampled metagenomes of the *Tara* Oceans expedition (Brum et al., 2015; Sunagawa et al., 2015). A recent study successfully detected viruses-host interactions by such an approach in samples from a freshwater lake (Okazaki et al., 2019). We also applied a protein homology-based method after carefully examining prediction parameters for prediction of Bacteroidetes viruses, which enabled a more sensitive signal detection than previously proposed nucleotide similarity-based *in silico* methods.

## Materials and Methods

### Collection of Viral and Bacteroidetes Genomes

We used the previously assembled 1,811 (EVGs; all being circularly assembled genomes) derived from marine viromes (Nishimura et al., 2017a). Genus-level genomic operational taxonomic units (gOTUs) were assigned to these EVGs as previously described (Nishimura et al., 2017a). We also collected 53 isolated dsDNA Bacteroidetes viral genomes and 100 randomly selected isolated prokaryotic viral genomes infecting non-Bacteroidetes prokaryotes (e.g., Proteobacteria) as reference viral genomic data from NCBI RefSeq (as of April 2019; Supplementary Table S1).

Bacteroidetes genomes that were publicly available prior to April 2019 were collected from NCBI RefSeq (total 3,695 genomes representing 2,148 species, Supplementary Table S1) and used as references for the host prediction analysis. We also collected 3,882 MAGs from the *Tara* Oceans metagenomic datasets (here after referred to as TARA-MAGs), which include 518 MAGs assigned to the phylum Bacteroidetes in the original studies (here after referred to as Bacteroidetes-MAGs, listed in Supplementary Table S1) (Tully et al., 2017, 2018; Delmont et al., 2018). To remove the contamination of virus-like contigs from TARA-MAGs, 11,537 contigs predicted as viral-like sequence (category 1, 2, and 3) by VirSorter (Roux et al., 2015a) were discarded from 1,732 MAGs (Supplementary Table S1). Taxonomy of the Bacteroidetes-MAGs predicted as hosts of EVGs were further confirmed based on the conserved maker genes in bacterial genomes by GTDB-Tk with classify mode (Chaumeil et al., 2019).

### Host Prediction by Nucleotide Similarity-Based Methods

We used four computational host prediction strategies that are frequently used to identify potential virus-host interactions. All of these methods utilize nucleotide sequence similarity for prediction, and details of these methods are reviewed elsewhere (Edwards et al., 2016). (i) CRISPR spacers match: CRISPR spacer sequences from Bacteroidetes genomes were predicted by CRISPR Recognition Tool (Bland et al., 2007). Sixty-nine thousand one hundred and seventy-two and 2,004 spacer sequences were extracted from the Bacteroidetes genomes in NCBI RefSeq and MAGs, respectively. Detected spacer sequences were queried against EVGs using the BLASTn-short function with these parameters: at least 95% identity over the whole spacer length and only 1–2 SNPs at the 5′ end of the sequence was allowed. (ii) tRNA match (Paez-Espino et al., 2016): tRNAs were recovered from bacterial genomes and EVGs by ARAGORN with “-t” option (Laslett and Canback, 2004). tRNAs (192,217, 13,018, and 6,322) were recovered from the Bacteroidetes genomes in NCBI Refseq, MAGs, and EVGs, respectively. The recovered tRNAs were compared by BLASTn (Camacho et al., 2009) and only a perfect match (100% length and 100% sequence identity) was considered indicative of putative Bacteroidetes-virus pairs. (iii) Nucleotide sequence homology of Bacteroidetes genomes and EVGs: EVGs were queried against Bacteroidetes genomes using BLASTn (Camacho et al., 2009). Only the best hits above 70% identity across alignment with length ≥1000 bp were indicative of Bacteroidetes-virus pairs. (iv) Oligonucleotide frequency (ONF) distance: Oligonucleotide frequency and distance between MAGs and EVGs were calculated by VirHostMatcher with a dissimilarity score <0.13 as an indication of Bacteroidetes-virus pairs (Ahlgren et al., 2017).

We performed taxonomic validation for each contig in Bacteroidetes-MAG showing similarity with EVGs in the above methods (CRISPR, tRNA, and nucleotide sequences homology) by the following procedures as previously described with slight modification (Coutinho et al., 2017). Open reading frames (ORFs) of each contig were predicted by MetaGeneMark with -p 0 option (Zhu et al., 2010) and queried against RefSeq database (as of May 2018) by BLASTp (*E*-value <1e-10, identity >30%, and bit score >50). The sum of the bit score of the all best hits from each contig was calculated, and if >80% of the total bit score was consistently assigned to Bacteroidetes, the contig of the MAG was considered to be derived from Bacteroidetes genomes; otherwise it was considered as a contaminant contig from other taxa (i.e., not Bacteroidetes). If a contig was regarded as contaminant contigs, the EVG showing similarity with the contig were removed from candidates of Bacteroidetes virus. Similarly, to remove viral contamination-like contigs in RefSeq Bacteroidetes genomes, the contigs predicted as viruses by VirSorter (Roux et al., 2015a) were discarded.

### Calculation of the Proportion of Bacteroidetes Homologs in Viral Genomes

ORFs for the viral genomes were predicted by MetaGeneMark with -p 0 option (Zhu et al., 2010). Homology search was conducted using BLASTp against the RefSeq database (as of May 2019, bit score >50). Similarly, BLASTp search was conducted against the ORFs of TARA-MAGs predicted by MetaGeneMark with -p 0 option (Zhu et al., 2010). Taxonomic validation to the matched contigs of the MAGs was performed as described in the previous section. Among the most closely matched cellular homologs of a viral genome, proportion of the Bacteroidetes homologs was calculated. To check the possible origin of the Bacteroidetes homologs, putative provirus regions in the Bacteroidetes genomes were checked by VirSorter (category 4, 5, and 6) (Roux et al., 2015a). If the Bacteroidetes homologs were encoded within the provirus region, the Bacteroidetes homologs were regarded as provirus origin.

### Proteomic Tree Calculation

The viral proteomic tree (Rohwer and Edwards, 2002) was calculated between 4,240 viral genomes in a previous study (Nishimura et al., 2017a) or constructed based on their genome similarity scores derived from all-against-all tBLASTx computation as previously described (Bhunchoth et al., 2016; Nishimura et al., 2017a, b). Parts of the proteomic tree were visualized from ViPTree webserver (Nishimura et al., 2017b) and an interactive visualization server of viral genomes developed in a previous study (Nishimura et al., 2017a^1^).

### Gene Prediction and Annotation

Gene prediction and functional annotation of the EVGs were obtained from a previous study (Nishimura et al., 2017a). Additionally, to explore the auxiliary metabolic genes (AMGs), ORFs were queried against the Pfam domain database v.31 (Finn et al., 2016) with hmmsearch (threshold 10^–5^ for *E*-value) (Eddy, 2011) and annotated by eggNOG-mapper (Huerta-Cepas et al., 2017) using eggNOG 5.0 database (Huerta-Cepas et al., 2019). Protein motifs found in the AMGs were defined according to previous studies (Roux et al., 2016; Luo et al., 2017).

### Phylogenetic Trees of Gp23 of Far-T4 Like Viruses

Far-T4 reference genomic fragments assembled from freshwater viromes were obtained from Metavir web server under project “FarT4/Far-T4 Lake Pavin” (Roux et al., 2015b). Other reference sequences were obtained from the NCBI RefSeq database of complete viral genomes. Multiple sequences were aligned using the MAFFT program (version 7.245) (Katoh et al., 2002), with the FFT-NS-2 mode and a maximum of 1,000 iterations (–retree 2, –maxiterate 1000). Conserved positions in the alignments were selected with the trimAl program (version 1.3) (Capella-Gutierrez et al., 2009). Approximately maximum likelihood trees were constructed by FastTree (Price et al., 2010) and visualized by iTOL (Letunic and Bork, 2019).

### Virome Read Mapping

Forty-three *Tara* Oceans viromes were downloaded from the European Nucleotide Archive^2^ under accession numbers reported in the original study (Brum et al., 2015) and quality control was performed as previously described (Nishimura et al., 2017a). The quality controlled sequences were mapped against the 1,811 EVGs using Bowtie2 with a parameter “–score-min L,0,-0.3” (Langmead and Salzberg, 2012). Fragments per kilobase per mapped million reads (FPKM) values were calculated by in-house ruby scripts (Nishimura et al., 2017a).

## Results

### Detection of Bacteroidetes Viruses by Nucleotide Similarity-Based Methods

To identify novel Bacteroidetes-virus pairs, we first conducted host prediction analyses on the 1,811 EVGs based on CRISPR spacer sequences, tRNA genes, sequence similarity (BLASTn) and ONF distance, by using 3,695 Bacteroidetes genomes in NCBI RefSeq and 518 Bacteroidetes-MAGs (Table 1). In total, we detected 57 signals of virus-host interactions between EVGs and Bacteroidetes-MAGs or Bacteroidetes genomes in RefSeq. An EVG (TARA_ERS490053_N000309) was predicted as Bacteroidetes virus with both datasets. After removal of redundancy, 35 EVGs including 18 previously described as members of *Flavobacteriaceae* viruses were predicted as putative Bacteroidetes viruses. Of these, OBV_N00073 and OBV_N00010 were previously predicted as viruses infecting SAR 11 and Marine group II archaea, respectively. We discarded these two EVGs as false positives from further analysis, taking into consideration the limitation of computational host prediction accuracy (Edwards et al., 2016) and the previous detailed analysis (Nishimura et al., 2017a). The remaining 33 Bacteroidetes EVGs were classified into 18 genus-level groups (gOTUs) based on the viral genome similarity (Supplementary Table S2)

**TABLE 1:** The number of EVGs assigned to Bacteroidetes viruses according to nucleotide based-methods (i.e., CRISPR, tRNA, BLASTn, and oligonucleotide frequency) using Bacteroidetes genomes.

			**BLASTn**	**Oligo nucleotide**	
	**CRISPR**	**tRNA**	**(>1 kb)**	**frequency**	**Total**
3,695 Refseq Bacteroidetes genomes	3	0	16	0	19
518 TARA Bacteroidetes MAGs	1	14	18	5	38

The nucleotide similarity-based approaches for the EVGs and Bacteroidetes genomes in RefSeq revealed 20 signals of virus-host interactions (between 6 EVGs and 18 Bacteroidetes genomes in RefSeq; Supplementary Table S2). All the 6 EVGs were classified as the members of the *Flavobacteriaceae* EVG group 1, previously identified by their genomic similarity to cultured Bacteroidetes viruses (Supplementary Table S2). Putative host Bacteroidetes of these EVGs were members of *Flavobacteriaceae* isolated from marine environments such as sea water (Nedashkovskaya et al., 2005; Yu et al., 2014; Gao et al., 2015; Xing et al., 2015), marine sediment (Miyazaki et al., 2010; Lee et al., 2014), sponges (Esteves et al., 2013; Jackson et al., 2015), and coral reef (Keller-Costa et al., 2016) samples. Our results not only support the previous host prediction studies based on genomic similarity with cultivated Bacteroidetes viruses and genomic context (Nishimura et al., 2017a), but also offer additional clues for lineage specific interaction between *Flavobacteriaceae* EVGs and Bacteroidetes. For example, two EVGs classified into a genus-level genomic OTU (G490 in the previous study, Nishimura et al., 2017a) were paired with *Aquimarina* species which is associated with marine sponge or coral reef (Supplementary Table S2).

The nucleotide similarity-based approaches for the EVGs and Bacteroidetes-MAGs revealed 37 signals between 26 EVGs and 13 MAGs (Supplementary Table S2). Although Bacteroidetes-MAG data were seven-folds smaller in size than the genomic data from RefSeq, Bacteroidetes-MAGs have twice as many significant signals with EVGs. Among the 26 putative Bacteroidetes EVGs, two and 11 EVGs were members of the *Flavobacteriaceae* EVG group 1 and group 2, respectively (Nishimura et al., 2017a). Also, TARA_ERS490388_N000065 showed nearly genus-level similarity with *Cellulophaga* viruses classified into Cba41likevirus (Holmfeldt et al., 2013). In addition to these previously described Bacteroidetes EVGs, we detected 12 new candidates of Bacteroidetes EVGs classified into five genus-level groups from MAG-based prediction (Supplementary Table S2). We performed taxonomic classification of the putative host MAGs by genome-based phylogeny (Parks et al., 2018). We could not classify some of these putative host MAGs because of the low completeness. However, the classification of high completeness MAGs suggests that most of the putative host MAGs are members of marine uncultured Bacteroidetes lineages, from which no viruses have been previously described (Supplementary Table S2). For example, three MAGs were classified into candidates genus SHAN690 mostly composed of marine MAGs (Parks et al., 2017) and one MAG was classified into another candidates genus MS024-2A mostly composed of marine single cell genomes (Woyke et al., 2009).

### Detection of Bacteroidetes Viruses by Protein Homology-Based Approach

The nucleotide similarity-based approaches enabled us to detect a large number of Bacteroidetes viruses when combined with the TARA-MAG data than when it was solely based on cultured strain genomes. However, most members of the previously described 239 *Flavobacteriaceae* EVGs were still not detected by the nucleotide similarity-based methods (Nishimura et al., 2017a). This was due to the fact that the nucleotide similarity-based prediction methods rely on rare and/or strain specific evolutionary events such as acquisition of CRISPR spacer or horizontal gene transfer (Edwards et al., 2016). Further, nucleotide sequence-based comparison can detect only recent evolutionary events because nucleotide sequences can change more rapidly than protein sequences because of redundancy in the genetic code (Edwards et al., 2016). We therefore developed a more sensitive method to detect Bacteroidetes viruses based on protein-homology. Bacterial homologes (i.e., the match with the lowest *E*-value) of viral encoded proteins are frequently found in Bacterial genomes in the same phylum as the host of the viruses (Mahmoudabadi and Phillips, 2018). Actually, 10–92% of proteins encoded in the genomes of the *Flavobacteriaceae* EVG groups 1 and 2 were most similar to Bacteroidetes genes (Nishimura et al., 2017a). However, the proportion of Bacteroidetes homologs was not tested in other EVGs and the prediction method was not standardized in the previous study. We hypothesized that the Bacteroidetes viruses have more Bacteroidetes homologs than other prokaryotic viruses, and thereby the proportion of Bacteroidetes homologs in viral genomes may be a useful genetic signal of Bacteroidetes viruses.

**FIGURE 1:**
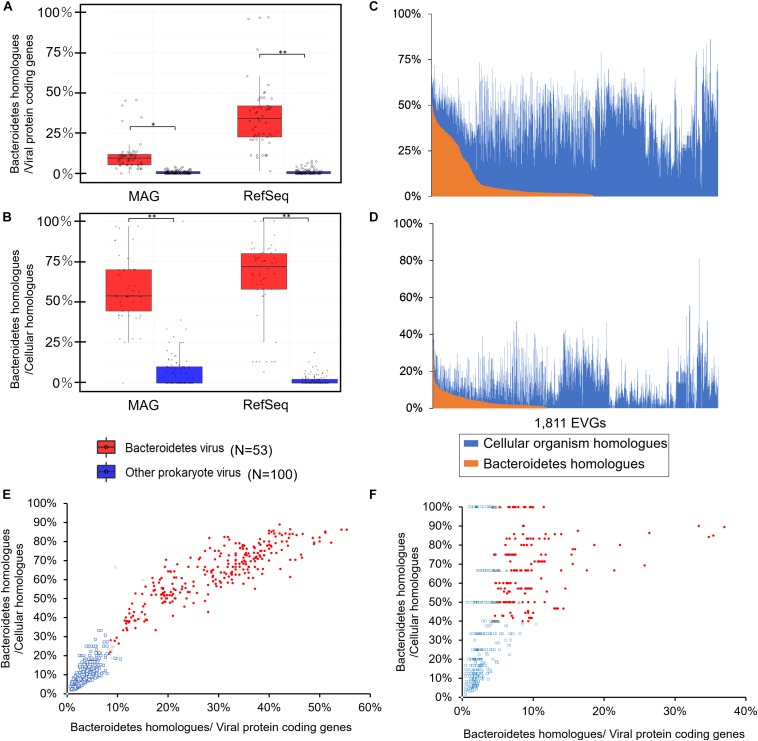
Proportion of the Bacteroidetes homologs in viral genomes. **(A)** Proportion of the Bacteroidetes homologs among protein-coding genes. **(B)** Proportion of the Bacteroidetes homologs among cellular organism homologs. The proportions on the right are those calculated from NCBI RefSeq and those on the left are calculated from TARA MAGs. Red and blue boxes represent cultured Bacteroidetes viruses and cultured viruses infecting other prokaryotes, respectively. The boxes represent the first quartile, median, and third quartile. Asterisks denote significance (Mann–Whitney *U*-test, *P < 0.05, **P < 0.001). **(C)** Proportion of the Bacteroidetes homologs in RefSeq (orange) and other cellular organism homologs in RefSeq (blue) of the 1,811 EVGs. **(D)** Proportion of the Bacteroidetes homologs in MAGs (orange) and other cellular organism homologs in TARA MAGs (blue) of the 1,811 EVGs. Scatter plots showing the proportion Bacteroidetes homologs among protein-coding genes (*x*-axis) and among cellular organism homologs (*y*-axis) for the comparison against RefSeq **(E)** and TARA-MAG **(F)**. Viruses passing the cut off values for the prediction of Bacteroidetes EVGs are shown in red circles. Viruses passing the two criteria (i.e., (i) at least 7.9% or 4.2% of genes should be homologs of Bacteroidetes genes in RefSeq or TARA-MAGs, respectively; (ii) the Bacteroidetes homologs should account for at least 18.8 or 38.9% of cellular homologs in RefSeq or TARA-MAGs, respectively) but have only few Bacteroidetes homologs (RefSeq: homolog <5 genes, TARA MAGs: homolog <3 genes) are shown in gray triangles. Other viruses that did not pass the cut off values are shown in blue squares.

Firstly, we examined the proportion of proteins that best hit to Bacteroidetes proteins (defined as the most similar protein detected by BLASTp; *E*-value <1e-10, identity >30%, and bit score >50) for cultured Bacteroidetes viruses (Figures 1A,B, and Supplementary Figure S1). As expected, most of the cultured Bacteroidetes viruses have many homologs of Bacteroidetes in RefSeq (average 35.8%) or TARA-MAGs (average 11.6%) in their genomes (Figures 1A,B, and Supplementary Figure S1). Among the possible homologs-sharing mechanisms between bacteria and viruses, we examined the contribution of provirus and AMGs to the shared homologs. Provirus-like regions in Bacteroidetes genomes appeared to mainly contribute (Supplementary Figure S2, average: 55.5%, maximum: 96%) to these homologs. This trend was observed not only in the lysogenic viruses or viruses having putative integrase homologs but also in the lytic Bacteroidetes viruses (Supplementary Figure S3). In contrast, AMGs rarely contributed (Supplementary Figure S2, average: 3%, maximum: 11%) to the detection of Bacteroidetes homologs (Supplementary Figure S2). The viruses infecting other prokaryotes (i.e., non-Bacteroidetes viruses) rarely showed Bacteroidetes homologs (Figures 1A,B, and Supplementary Figure S1, at most 7.9 and 4.2% to Bacteroidetes in RefSeq and TARA-MAGs, respectively). According to the comparison of the result between Bacteroidetes viruses and non-Bacteroidetes viruses, we chose the following criteria for the prediction of putative Bacteroidetes EVGs. We considered EVGs that satisfy all the following three criteria as Bacteroidetes EVGs: (i) At least 7.9 or 4.2% of viral genes should be homologs of Bacteroidetes genes in RefSeq or TARA-MAGs, respectively, (ii) The Bacteroidetes homologs should account for at least 18.8 or 38.9% of cellular homologs in RefSeq or TARA-MAGs, respectively, and (iii) At least 5 or 3 viral genes should be Bacteroidetes homologs in RefSeq or TARA-MAGs, respectively. Each threshold corresponds to the maximum value observed for non-Bacteroidetes viruses.

By applying these criteria to 1,811 EVGs, we identified 311 EVGs as putative Bacteroidetes viruses (Figures 1C–F). All of the 239 EVGs that were previously described as members of *Flavobacteriaceae* group 1 and 2 (Nishimura et al., 2017a) were included in these putative Bacteroidetes EVGs. Seventy-two EVGs were newly predicted as Bacteroidetes viruses.

### Classification of Bacteroidetes EVGs and Their Genomic Features

There are 21 overlaps between the Bacteroidetes EVGs predicted based on nucleotide similarity-based methods and protein homology-based method (Supplementary Figure S3). Ten EVGs were only predicted by nucleotide similarity-based methods using MAGs and 290 EVGs were only predicted by protein homology-based method (Supplementary Figure S3). In total, we identified 321 EVGs as putative Bacteroidetes EVGs including 81 EVGs which were not predicted as their host in previous studies. The 321 EVGs were classified into 29 gOTUs based on their genomic similarity (Nishimura et al., 2017a, Supplementary Figure S4). In the following sections, we describe the genomic features of 81 EVGs, which are the newly identified putative Bacteroidetes viruses.

### Novel Sub-Clade of Flavobacteriaceae EVG Group 1

Twenty-four EVGs of two gOTUs (G493 and G494) were located near the branches of the previously described *Flavobacteriaceae* EVGs group 1 in the viral proteomic tree (Supplementary Figure S4). These EVGs were 27.5–50.5 kb with an average G+C content of 32.6% (Table 2). Putative viral structural protein genes (major capsid, prohead protease, terminase, and portal) and putative DNA replication genes were well conserved within the viral group. Genome synteny of the tail like structure such as putative endosialidase tail spikes were also conserved but exhibited low sequence homology within the group (Supplementary Figure S5A). They also shared portal gene homologs conserved in the members of the group 1 (Supplementary Figure S5A). Therefore, we concluded that the twenty-four EVGs are new members of the subclade of *Flavobacteriaceae* EVGs group 1.

**TABLE 2:** General genomic features of the Bacteroidetes gOTUs identified in this study.

**Group (gOTU)**	**No of EVGs**	**No. of EVGs predicted as Bacteroidetes EVG**	**Ave. length (bp)**	**Ave. GC%**	**Refseq Bacteroidetes homologe in EVG (Ave).**	** MAG Bacteroidetes homologe in EVG(Ave).**	**Classified group**
G160	13	9	37,551	38.5	2.2%	11.1%	
G178	1	1	40,754	32.6	11.4%	0%	
G185	4	2	54,812	31.7	1.9%	0.5%	
G189	3	1	58,769	35.4	5.3%	3.7%	
G199	2	1	36,245	35.8	5.9%	2.5%	
G203	5	2	31,173	30.7	5.7%	7.0%	
G204	3	3	32,490	32.4	4.4%	6.4%	
G205	2	2	27,613	33.8	3.5%	15.1%	
G206	4	3	27,672	35.8	3.2%	7.4%	
G207	3	1	31,013	33.2	4.7%	4.7%	
G210	8	5	34,852	38.2	7.5%	4.6%	
G211	1	1	34,002	34.9	7.8%	9.8%	
G341	1	1	39,514	39.3	10.2%	10.2%	
G398	1	1	179,949	32.0	6.3%	0.4%	T4 like
G405	1	1	143,709	33.4	8.5%	7.3%	Far-T4 like
G493	21	21	32,686	33.5	31.8%	5.3%	Novel sub-clade of Flavobacteriaceae group 1
G494	3	3	31,174	31.7	22.8%	8.0%	
G535	1	1	33,328	30.5	36.0%	4.0%	
G536	1	1	39,973	35.3	28.6%	7.1%	*Flavobacteriaceae* EVGs group 3
G537	1	1	41,032	42.0	55.4%	21.4%	
G541	4	4	33,608	40.6	43.6%	35.1%	
G542	1	1	44,120	33.1	36.1%	22.2%	
G544	1	1	38,581	32.6	44.1%	8.5%	
G561	1	1	42,760	32.6	25.8%	1.6%	Bacteroidetes viral lineages
G563	1	1	51,661	49.2	3.3%	4.9%	
G790	1	1	58,364	33.9	34.7%	26.4%	
G794	9	7	12,003	31.2	0%	0%	
G810	3	1	43,470	46.8	2.0%	2.3%	
G815	3	3	32,908	39.6	28.6%	30.8%	

### Flavobacteriaceae EVGs Group 3

We detected a novel group (group 3) of putative marine *Flavobacteriaceae* viral genomes (Supplementary Figure S4B). This group composed of 10 EVGs classified into 5 gOTUs and 19 cultured Bacteroidetes virus genomes. The 10 EVGs ranged in size from 32–44 kb with a G+C content ranging from 32.6–42% (Table 2). The EVGs shared 2.8–30.4% of genes (two to seven genes) with the cultured members of the group 3. For example, TARA_ERS492198_N000180 (G537) and TARA_ERS490204_N000278 (G536) shared 17 and 8 genes with *Cellulophaga* siphovirus phi19:1, respectively (Supplementary Figure S5B). Most of the shared genes are annotated as structural protein genes such as capsid and tail tape measure (Supplementary Figure S5B). However, the EVGs rarely shared genes with *Cellulophaga* siphovirus phi10:1, which show genus level similarity with phi19:1 (Supplementary Figure S5B). Similarly, within the group 3, LDNO01000008 and *Flavobacterium* virus 11b shared several structural protein homologs with phi10:1 but not with phi19:1 or the members of G537 and G536 (Supplementary Figure S5B).

### Other Bacteroidetes Viral Lineages

We identified two other EVGs (TARA_ERS491107_N000194 and LDNO01000002) positioned within a clade of the proteomic tree exclusively composed of Bacteroidetes viruses infecting members of *Flavobacteriaceae* and *Bacteroides* (Supplementary Figure S5C). The two EVGs had 42–51 kb genomes with a G+C content of 32.6 and 49.2%, respectively (Table 2). TARA_ERS491107_N000194 shared a maximum 21% of the genes (17 genes) including putative capsid protein genes and phage tail protein gene with the cultured members of this group. Similarly, LDNO01000002 shared maximum 5% of the genes (two genes) such as putative terminase-like protein with the members of this group but did not share any genes with TARA_ERS491107_N000194.

### T4 Like Viruses

**FIGURE 2:**
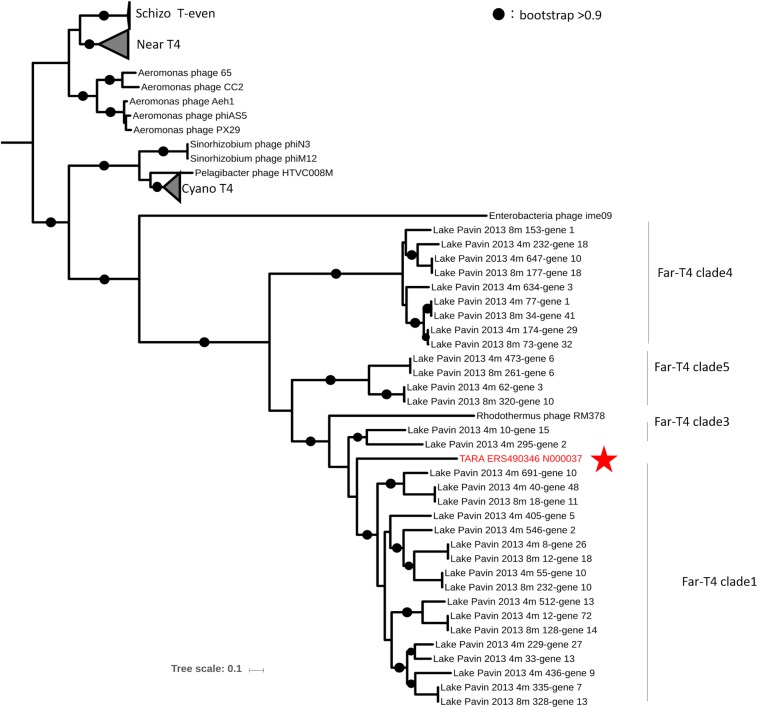
An approximately maximum likelihood phylogenetic tree computed from the multiple alignment of Gp23 (major capsid protein) of TARA_ERS490346_N000037 (G405) and T4-like superfamily viruses. The protein sequences were collected from RefSeq and Lake Pavin viromes (Roux et al., 2015b). Circles indicate nodes with bootstraps higher than 0.9.

We identified two EVGs which exhibited genome characteristics of the T4-like superfamily (*Tevenvirinae*), which is one of the most widespread, abundant, and extensively studied viral groups. This is the first report of T4-like viruses infecting marine Bacteroidetes excluding a virus infecting thermophilic Bacteroidetes *Rhodothermus marinus*. *Tevenvirinae* appears to be comprised of several subgroups including (i) the “true” T-evens represented by T4 and closely related viruses infecting Enterobacteria, (ii) the Pseudo and Schizo T-evens (including Aeromonas and Vibrio viruses), (iii) the Exo T-evens (including cyano- and SAR11 viruses), and Far-T4-like virus, which includes the sole isolate RM378 infecting a thermophilic Bacteroidetes *R. marinus* (Petrov et al., 2010). TARA_ERS490346_N000037 (G405), 143 kb in size with a G+C content of 33.4% (Table 2) was found to be most similar to the Far-T4-like virus RM378 among the cultured viruses as they shared 26 genes (Supplementary Figure S6A). Phylogenetic tree of the major capsid protein (T4 phage gene 23) suggests that TARA_ERS490346_N000037 is a novel member of Far-T4 like viruses (Figure 2). This EVG is the first representative of complete genomes from environmental Far-T4 like virus with *in silico* identification of putative host groups. The EVGs have up to 66 genes mostly annotated as structural proteins and replication proteins shared with the Far-T4 genome fragments assembled from the freshwater viromes (Supplementary Figure S6A) (Roux et al., 2015b).

TARA_ERS488589_N000003 (G398) was observed to be most similar to the marine Exo-T4 like viruses infecting *Pelagibacter* and unicellular Cyanobacteria (Supplementary Figure S6B). This EVG has a 180 kb genome and G+C content is slightly lower (32%) than the known T4-like viruses (Table 2). Twenty two of the 38 core genes conserved in the T4-like virus genomes as shown in a previous comparative genomics study (Supplementary Figure S6B) (Sullivan et al., 2010).

As reported in the other T4 like viruses, these T4 like EVGs encoded putative auxiliary metabolic genes (Supplementary Table S3). For example, the TARA_ERS488589_N000003 has queuosine (Que) biosynthesis pathway genes [gene109 (*queF*), gene162 (*queE*), gene164 (*queD*), and gene66 (GTP cyclohydrolase)]. Que biosynthesis genes were reported in two cultured *Cellulophaga* viruses (Holmfeldt et al., 2013) and we found them in members of the *Flavobacteriaceae* group 1 and group 2 (Supplementary Table S3). Similarly, both EVGs encode proteins putatively related to carbohydrate metabolism (Supplementary Table S3). Additionally, we found that the TARA_ERS488589_N000003 encodes proteins putatively related to two cell-surface adhesion systems [curli biosynthesis (gene_61: *csrA*, gene_62: *csrG*, and gene_63; *csrF*)] and ubiquitous surface proteins (gene_52 and gene_70, Supplementary Table S3) mostly found in pathogenic bacteria (Barnhart and Chapman, 2006; Tong Tan et al., 2019).

### Other New Lineages Distant From the Cultured Viruses

The remaining 44 EVGs classified into 17 gOTUs were 12–59 kb in size with a G+C content of 31–47% (Table 2). They were distributed in twelve clades in the viral proteomic tree exclusively composed of EVGs (Nishimura et al., 2017a). Following the previous classification of 2,429 cultured prokaryotic viral genomes, gOTUs classification based on genomic similarity reflected the phylum-level host taxonomy with only two exceptions (Nishimura et al., 2017a). This suggests that the 22 EVGs, which were not predicted as Bacteroidetes viruses by the *in silico* virus-host prediction employed in the present study but were classified into the same gOTUs as Bacteroidetes EVGs, are also likely to be candidates of Bacteroidetes viruses (Table 2). Most of the predicted genes (71–94%) of these uncultured clades were functionally annotated as hypothetical proteins, as is common for environmental viruses (Seguritan et al., 2003; Borriss et al., 2007). The predicted functions/categories of the annotated genes were DNA metabolism (48%, the values provided here are averages), viral structural genes (21%), and host lysis (15%).

### Abundance and Distribution of the Bacteroidetes EVGs

**FIGURE 3:**
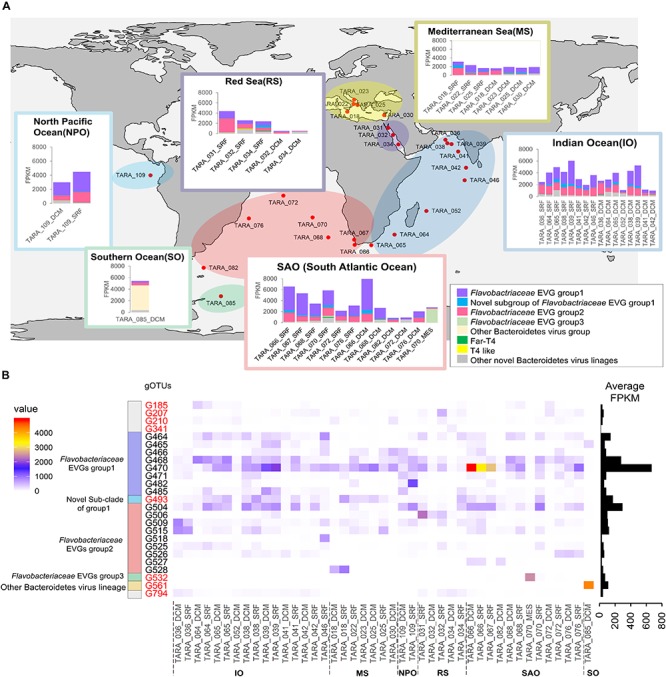
Abundance of the Bacteroidetes EVGs in Global Ocean surface waters. **(A)** Virome fragment recruitments of Bacteroidetes EVG groups in each oceanic region. Sampling sites of *TARA* ocean expedition used for analysis are shown in red circle. Bar graphs represents normalized virome FPKM (fragments per kilobase per mapped million reads) of each Bacteroidetes EVG group at the site. **(B)** A heatmap shows normalized virome FPKM of abundant Bacteroidetes EVGs (i.e., gOTUs passing average relative abundance >0.1% and/or relative abundance >15% at least a site within Bacteroidetes EVGs). The scale bar on the left side represents FPKM value. Average FPKM values are shown in the right panel. Novel Bacteroidetes EVGs detected in this study are highlighted in red text. Oceanic regioown in the map in panel **(A)** are shown under *x*-axis.

Abundance and distribution of the Bacteroidetes EVGs in the global ocean were investigated by read recruitment of the *Tara* Oceans viromes, which consist of 43 viromes representing 26 oceanic locations (Brum et al., 2015). Relative abundance of Bacteroidetes EVGs among the 1,811 EVGs ranged from 2.2–34.6% (average: 13.9%). Members of the *Flavobacteriaceae* EVGs group 1 were abundant along with the *Flavobacteriaceae* EVGs group 2, which includes phi38:1 belonging to one of the most abundant viral candidate genera in the global oceans (Roux et al., 2016). Most of the newly detected Bacteroidetes EVGs were less abundant (average: 2.8%) than *Flavobacteriaceae* EVG group 1 or 2 (average: 11%). However, members of the G493 were ubiquitous and fourth most abundant genus among the Bacteroidetes EVGs (Figure 3 and Supplementary Figure S7). Additionally, TARA_ERS491107_N000194 (G561) was rarely recruited reads from most samples but found to be locally abundant (up to 20% of the relative abundance) in the Chile-Peru Current Coastal Province deep chlorophyll maximum sample (Figure 3 and Supplementary Figure S7).

## Discussion

As only limited lineages of marine Bacteroidetes can be cultivated (Alonso et al., 2007), most viruses infecting marine Bacteroidetes have not been characterized. The objective of the study was to expand the knowledge of the diversity of the viruses likely infecting marine Bacteroidetes species by nucleotide/protein similarity-based approaches using MAGs as well as isolated bacterial genomes.

Firstly, we showed that Bacteroidetes MAGs from *Tara* Oceans data serve as more sensitive references for the host prediction of the uncultured marine Bacteroidetes viruses as compared to the genomes in the public database mostly derived from cultured bacteria (Table 1). This high sensitivity of MAGs obtained from simultaneously sampled metagenomes with EVGs supports ecosystem specific interactions of Bacteroidetes and these viruses. Taxonomic assignment of the Bacteroidetes MAGs suggests that these are representative genomes of previously uncultured marine Bacteroidetes lineages (Supplementary Table S2). It strengthened our hypothesis that viruses of unknown hosts interact with uncultured bacteria and MAGs enabled us to detect potential interactions by overcoming the cultivation bias. However, it should be noted that MAGs likely include several contaminations of contigs from other taxa or viruses. Therefore, it is important to be careful of the pre-filtering steps such as removal of virus-like contigs and contaminated contigs of other taxa. Moreover, not only the MAGs, we identified several virus-like sequence contaminations from the reported Bacteroidetes genomes in NCBI RefSeq database. For example, we found that an 18.8 kb of circular contig from *Non-labens* sp. 1Q3 (Accession: NZ_RMVE00000000) shows 98.7% nucleotide identity to Cyanophage P-TIM40 across 98% of the region. Pre-filtering by viral detection tools such as VirSorter (Roux et al., 2015a) was also important for the accurate host prediction of viruses using cultivated bacterial genomes.

Secondly, we developed a protein homology-based host prediction approach. The approach achieved significant improvement of the detection of Bacteroidetes viruses compared to the nucleotide similarity-based approaches. High proportion of the host homologs likely derived from proviruses suggest that the methods mainly rely on the viral lysogeny (Supplementary Figure S3). The observation that most of the viral genomes of cultured Bacteroidetes have a number of provirus homologs implies that lysogeny may be a widespread feature in Bacteroidetes viruses and these proviruses are maintained in host genomes. This feature might be related to a copiotrophic and r-strategist lifestyle of cultivated species of coastal Bacteroidetes (Lauro et al., 2009). Relatively large host genomes are capable of maintaining proviruses because of the weak selective pressure from genome streamlining (Lauro et al., 2009). Additionally, the viral lysogenic potential might be adaptive to respond to the multifold change of host abundance during and after phytoplankton bloom (Teeling et al., 2012). The fact that lysogens are widespread (25–50% of the microbial genomes) in marine environments (Howard-Varona et al., 2017) suggests that the homolog-based approach may be applicable not only for Bacteroidetes viruses but also for the environmental viruses infecting other prokaryotes. Indeed, the possession of many host-related homologs was also reported in uncultured viruses potentially infecting the marine group II (MGII) euryarchaeota (Nishimura et al., 2017a). However, viruses infecting extremophile Bacteroidetes have fewer Bacteroidetes homologs than the other Bacteroidetes viruses (Rhodothermus virus RM378: 1.4%, Salisaeta icosahedral virus: 6.6%). One possibility is that the shortage of genomes of the extremophile microorganisms due to sampling bias caused fewer matches with the host like homologs in their viruses. Expansion of microbial genomes could assist in more precise and sensitive host prediction of uncultured viruses by the homolog proportion-based method.

The Bacteroidetes EVGs identified by these new approaches may provide useful genetic markers for studying viral importance in the ecological study of marine Bacteroidetes, such as viral roles in the rapid succession of various Bacteroidetes species during bloom (Teeling et al., 2012; Needham and Fuhrman, 2016). For example, G493 is the fourth most abundant marine Bacteroidetes virus in the genus-level and might have a large impact on the dynamics of the uncultured marine Bacteroidetes populations. Among these newly identified Bacteroidetes EVGs, we identified not only the relatives of cultured marine Bacteroidetes viruses, but also marine viral lineages phylogenetically distinct from the cultured marine Bacteroidetes viruses.

We detected potential virus-host interactions between marine Bacteroidetes and Far-T4 viruses. They were previously reported to be common in aquatic environments but data on their complete genomes are unavailable and they are not linked with their hosts (Roux et al., 2015b). As members of Bacteroidetes are also common in aquatic environments (Kirchman, 2002; Pommier et al., 2007), they are reasonable hosts of the uncultured Far-T4 lineages. These findings may provide important insights into the unknown ecology of Far-T4 viruses. Among the Far-T4 Bacteroidetes EVGs, we found several previously reported AMGs putatively related to carbohydrate metabolism, sulfur metabolism, and queuosine synthesis (Supplementary Table S3). Among them, queuosine synthesis genes were widely found in Bacteroidetes EVGs (T4 like Bacteroidetes EVG, member of *Flavobacteriaceae* EVGs group 1 and 2, Supplementary Table S3). Queuosine is a hypermodified guanosine derivative in tRNAs specific for Asp, Asn, His, or Tyr. One of the predicted roles of queuosine is the improvement of translation efficiency (El Yacoubi et al., 2012) and a study suggested that it acts as a quantity control mechanism of viral structural gene products (Sabri et al., 2011). Other studies suggest queuosine modification of viral DNA provides a protection mechanism against host endonucleases (Kulikov et al., 2014; Thiaville et al., 2016; Sazinas et al., 2018). The biological role of queuosine modification is still controversial (Vinayak and Pathak, 2009); however, the prevalence of queuosine synthesis potential in marine Bacteroidetes EVGs suggests its advantage to the viruses during infection in marine Bacteroidetes. Additionally, we found two systems putatively related to cell adhesion (curli production and ubiquitous cell surface proteins) in an EVG (Supplementary Table S3). Curli amyloid fiber is a major proteinaceous component of the extracellular matrix produced mainly by Enterobacteriaceae (Barnhart and Chapman, 2006) and was also reported in Bacteroidetes genomes by bioinformatic analysis (Dueholm et al., 2012). The ubiquitous surface proteins are essential for the attachment of pathogenic *Moraxella* (Lafontaine et al., 2000; Tan et al., 2005). The genes might promote the attachment of infected host cells near the uninfected host cells during infection. Such aggregation during infection was recently reported in Tupanvirus infecting amoebas and thought to promote progeny production (Oliveira et al., 2019). Further studies are needed to clarify the role of these proteins in the life cycle of the EVGs.

## Conclusion

From the analysis of the host prediction of 1,811 circular complete genomes, we detected 321 viral genomes that most likely correspond to Bacteroidetes dsDNA viruses. Microbial MAGs have advantages in the computational detection of uncultured marine Bacteroidetes viruses compared with the microbial genomes in the current public databases. We also developed a sensitive method for predicting Bacteroidetes viruses based on bacterial homolog detection in viral genomes. This enhanced prediction approach using MAGs and homolog detection tested on the marine Bacteroidetes-virus systems might be applicable for the host prediction of diverse uncultured viral genomes and might also expand the realm of characterized viruses in various environments. The newly identified Bacteroidetes EVGs expanded our knowledge of the marine Bacteroidetes viruses such as identification of interactions between aquatic ubiquitous viral group Far-T4 and marine Bacteroidetes. They may serve as useful genetic markers for the future studies on the interactions between Bacteroidetes and their viruses.

## Data Availability Statement

Publicly available datasets were analyzed in this study. This data can be found here: ftp://ftp.genome.jp/pub/db/community/EVG2017.

## Author Contributions

KT performed the analysis and prepared the manuscript. DM, YN, and HO contributed to the analysis, discussion, and preparation of the manuscript. TY contributed to the research design, results, discussion, the manuscript revision, and overall support for this study.

## Conflict of Interest

The authors declare that the research was conducted in the absence of any commercial or financial relationships that could be construed as a potential conflict of interest.

## Footnotes

1 https://www.genome.jp/tools/mg_viewer2/

2 www.ebi.ac.uk/ena/
