# Supplementary material for: In silico Prediction of Virus-Host Interactions for Marine Bacteroidetes With the Use of Metagenome-Assembled Genomes
Source: Front Microbiol. 2020 Apr 28;11:738. doi: 10.3389/fmicb.2020.00738 (PMC7198788; doi:10.3389/fmicb.2020.00738)
Supplement: Supplementary file 1 [file Data_Sheet_1.pdf]

Supplementary Material

In silico prediction of virus-host interactions for marine Bacteroidetes with the use of metagenome-assembled genomes

Kento Tominaga, Daichi Morimoto, Yosuke Nishimura, Hiroyuki Ogata, Takashi Yoshida

*** Correspondence:** Dr. Takashi Yoshida yoshida.takashi.7a@kyoto-u.ac.jp

Supplementary Table S1. List of bacterial genomes for the host prediction analysis and cultivated viral genomes used as reference data set.

Presented as a separate MS Excel file.

Supplementary Table S2. Predicted virus-host pairs between EVGs and Bacteroidetes genomes.

Presented as a separate MS Excel file.

Supplementary Table S3. List of eggNOG and PFAM domains annotation of the putative AMGs found in Bacteroidetes EVGs.

Presented as a separate MS Excel file.

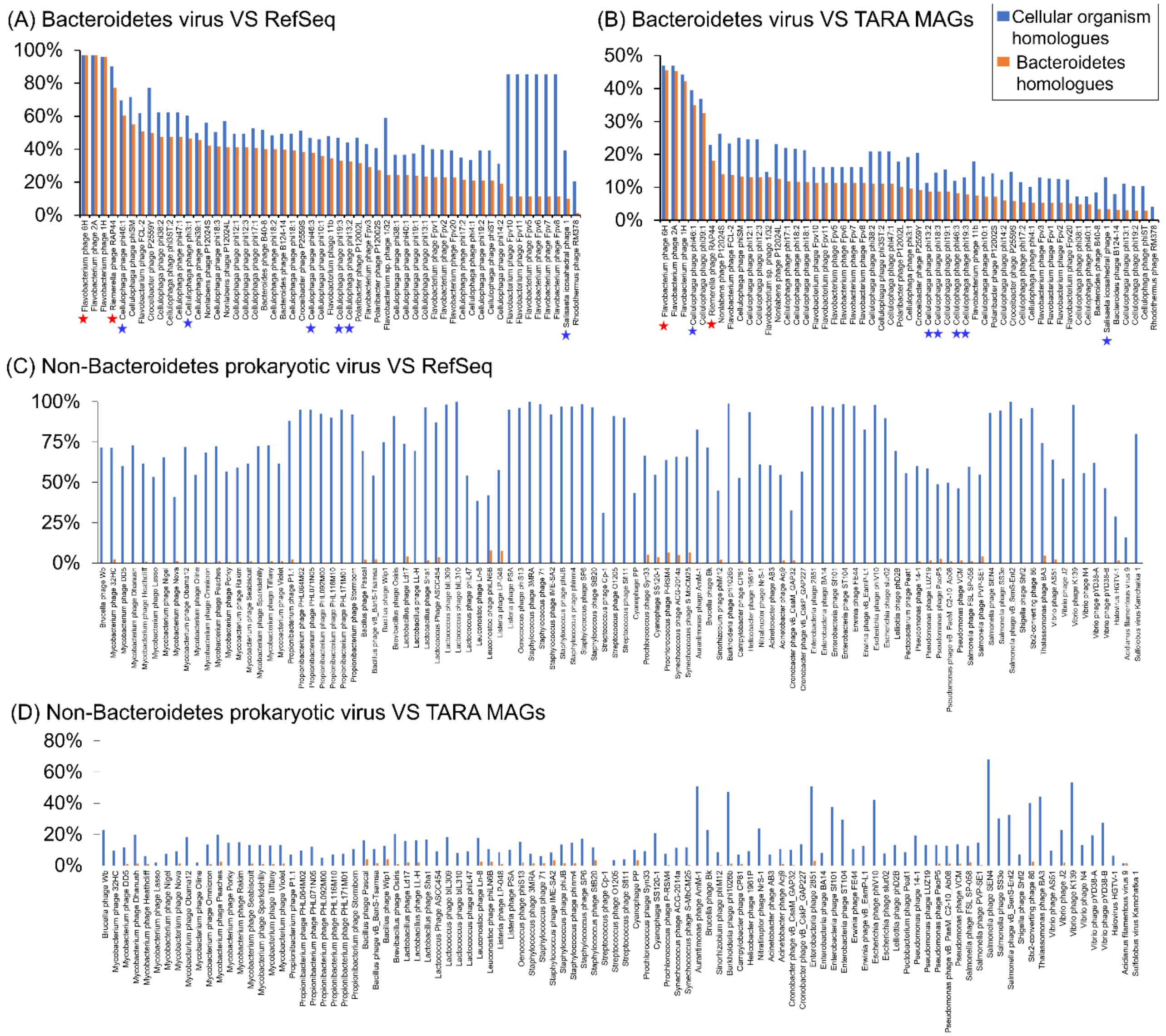

Supplementary Figure 1. Proportion of the Bacteroidetes homologs in cultivated viruses. (A) Proportion of the Bacteroidetes homologs in RefSeq (orange) and cellular organism homologs in RefSeq (blue) of the 53 dsDNA cultivated Bacteroidetes viruses. (B) Proportion of the Bacteroidetes homologs in TARA MAGs (orange) and cellular organism homologs in TARA MAGs (blue) of the 53 dsDNA cultivated Bacteroidetes viruses. Red and blue stars represent the viruses with a lysogenic life cycle and viruses having putative integrase homologs but not reported lysogenic life cycle, respectively. (C) Proportion of the Bacteroidetes homologs in RefSeq (orange) and cellular organism homologs in RefSeq (blue) of the randomly selected 100 prokaryotic viruses. (D) Proportion of the Bacteroidetes homologs in TARA MAGs (orange) and cellular organism homologs in TARA MAGs (blue) of the randomly selected 100 prokaryotic viruses.

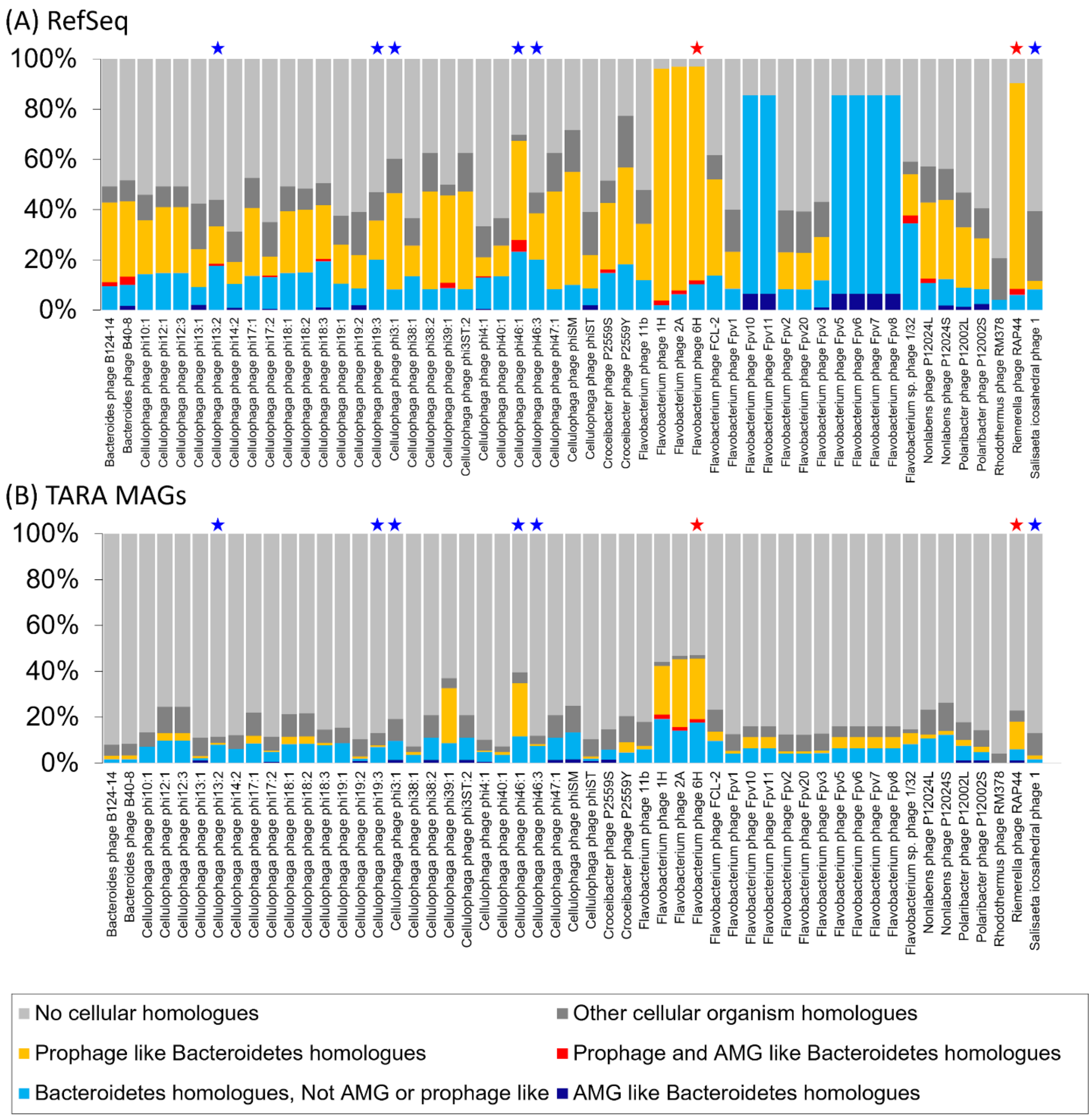

Supplementary Figure 2. Proportion of the putative AMGs and provirus homologs in the viral Bacteroidetes homologs. (A) Homologs of RefSeq cellular organism genomes. (B) Homologs of TARA MAGs. Proviruses were detected by VirSorter (Roux *et al.*, 2015). List of Pfam domains found in putative AMGs like genes followed the lists in Roux *et al.*, 2016 and Ruo *et al.*, 2018. Red and blue stars represent the viruses with a lysogenic life cycle and viruses having putative integrase homologs but not a lysogenic life cycle, respectively.

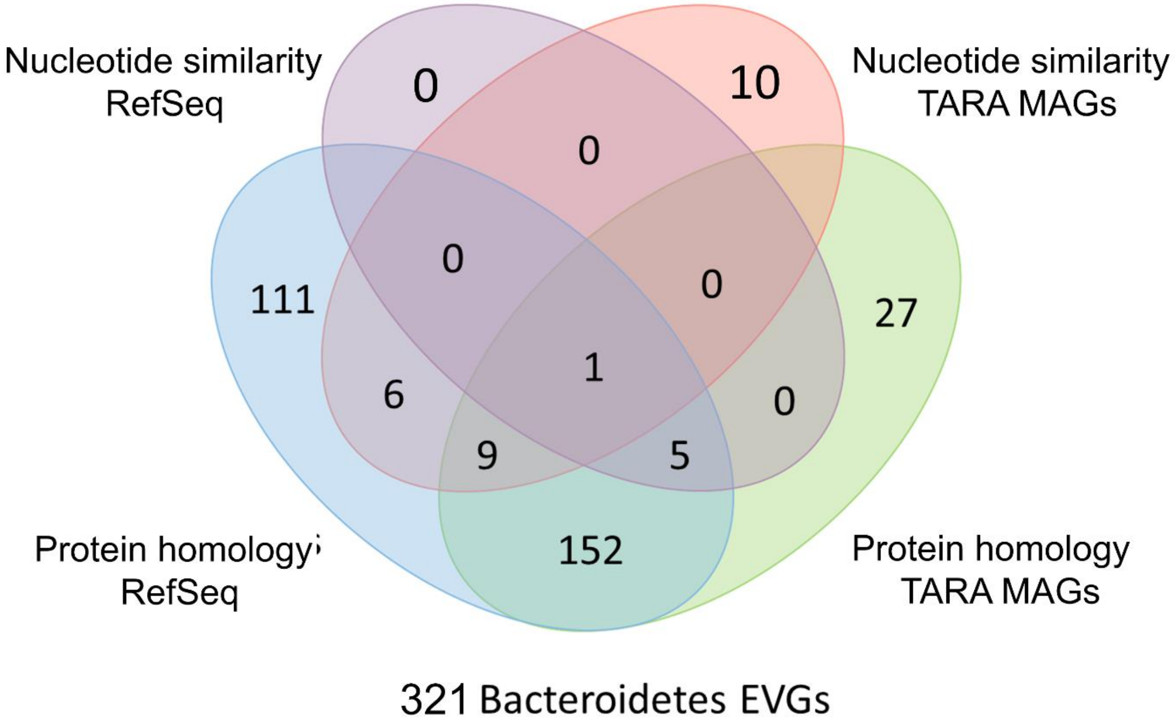

Supplementary Figure 3. Venn diagram of the Bacteroidetes EVGs detected by the four host prediction methods

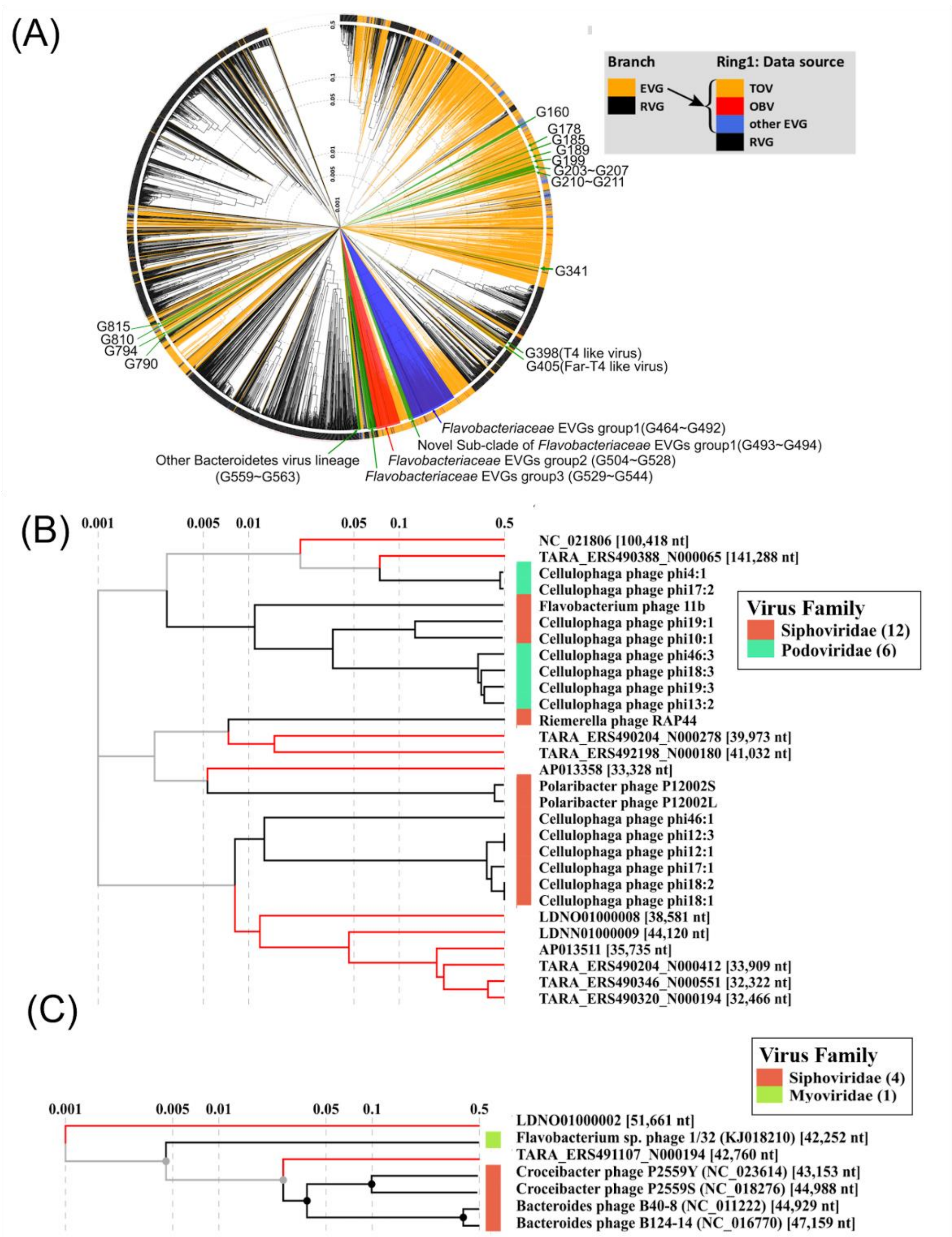

Supplementary Figure S4. Proteomic tree representation of the Bacteroidetes EVGs with cultured Bacteroidetes viral genomes. The dendrograms represent proteome-wide similarity relationships. (A) A proteomic tree of 1,811 EVGs (orange branches) and 2,429 cultured viruses (black branches) calculated in Nishimura *et al* 2017a with highlighting of newly detected Bacteroidetes EVGs (green), *Flavobacteriaceae* EVGs group1 (blue), and *Flavobacteriaceae* EVGs group2 (red). The tree is midpoint rooted. Branch lengths are indicated using a logarithmic scale. (B) A part of the proteomic tree with *Flavobacteriaceae* EVGs Group3 (red branches) and their relatives of cultured viruses (black branches). (C) A part of the proteomic tree with 2 EVGs (red branches) with cultured Bacteroides and *Flavobacteriaceae* viruses (black branches). Rings outside the dendrogram represent taxonomic groups of viral family classifications.

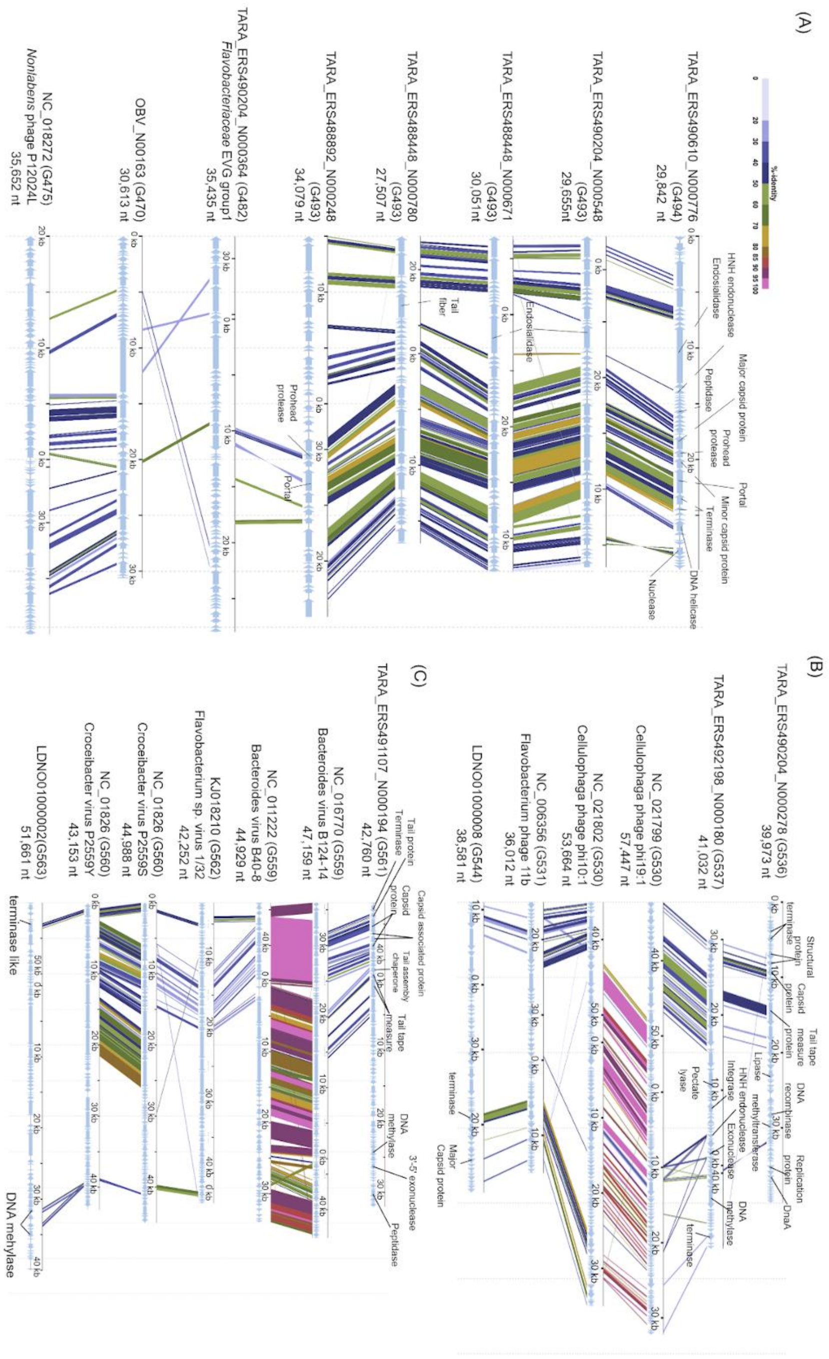

Supplementary Figure S5. Bacteroidetes EVGs shared genomic features with cultured Bacteroidetes genomes. (A) A genome map of members of the G493 and G494 with other members of the *Flavobacteriaceae* EVGs group 1. (B) A genome map of members of *Flavobacteriaceae* EVGs group 3. (C) A genome map of members of G561 and G563 with *Bacteroides* and *Flavobacteriaceae* viruses. The sequences are circularly permuted and/or reversed. The sequences are circularly permuted and/or reversed for clarity. Putative gene functions are indicated. All tBLASTx alignments are represented as colored lines between the two genomes. The color scale represents tBLASTx percent identity.

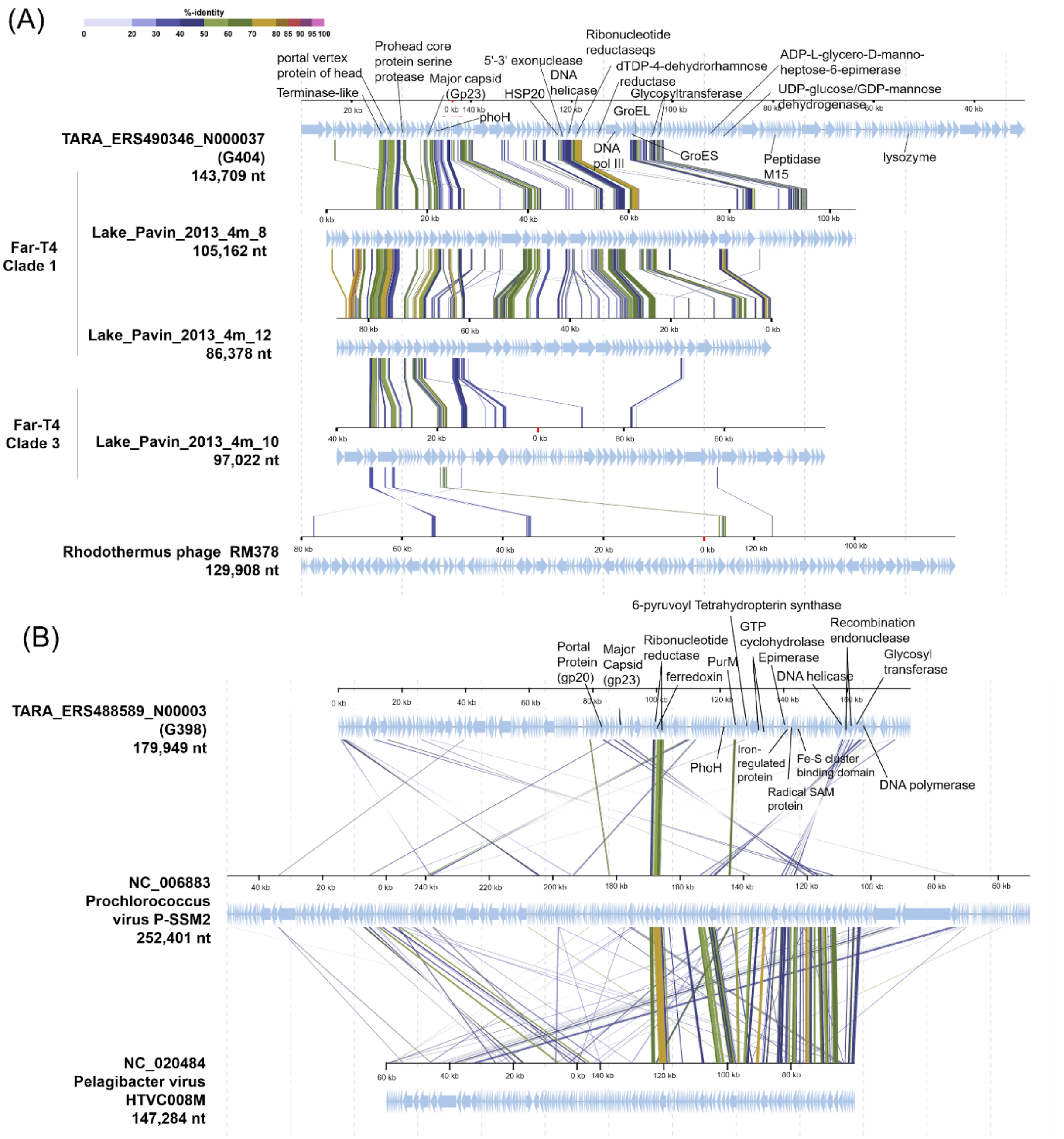

Supplementary Figure S6. Bacteroidetes EVGs shared genomic features with T4-like super family. (A) A genome map of TARA_ERS490346_N000037 (G405), Far-T4 contigs assembled in Roux *et al.* 2015 and *Rhodothermus marinus* virus RM378 (B) A genome map of TARA_ERS488589_N000003 (G398) and Exo-T4 viruses. The sequences are circularly permuted and/or reversed for clarity. Putative gene functions are indicated. All tBLASTx alignments are represented as colored lines between the two genomes. The color scale represents tBLASTx percent identity.

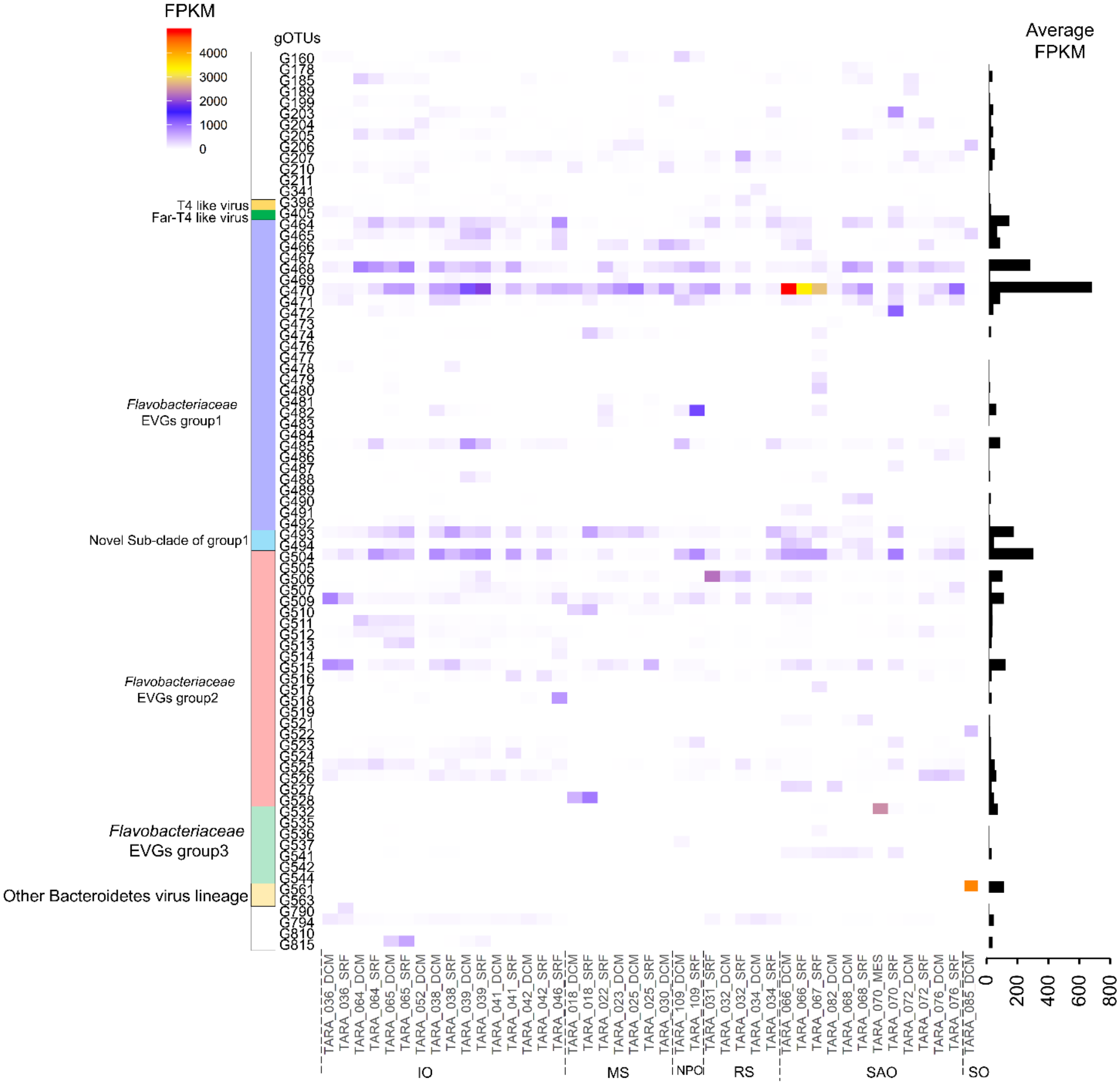

Supplementary Figure S7. Abundance of the Bacteroidetes EVGs in Global Ocean surface waters

A heatmap shows normalized virome FPKM (fragments per kilobase per mapped million reads) of the 83 genus-level OTUs including 322 Bacteroidetes EVGs. The scale bar on the left side represents FPKM value. Average FPKM values are shown in the right panel. X-axis represents sampling sites of *TARA* ocean expedition and oceanic region are abbreviated as: IO (Indian Ocean), MS (Mediterranean sea), NPO (North pacific ocean), RS (Red sea), SAO (South Atlantic Ocean), and SO (Southern Ocean).
